# Degree of Suppression of Mouse Myoblast Cell Line C_2_C_12_ Differentiation Varies According to Chondroitin Sulfate Subtype

**DOI:** 10.3390/md14100193

**Published:** 2016-10-21

**Authors:** Katsuhiko Warita, Nana Oshima, Naoko Takeda-Okuda, Jun-ichi Tamura, Yoshinao Z. Hosaka

**Affiliations:** 1 Veterinary Anatomy, Department of Veterinary Science, Faculty of Agriculture, Tottori University, Tottori 680-8553, Japan; waritak@muses.tottori-u.ac.jp (K.W.); hsk7030@gmail.com (N.O.); 2 Laboratory of Basic Veterinary Science, United Graduate School of Veterinary Science, Yamaguchi University, Yamaguchi 753-8515, Japan; 3 Department of Regional Environment, Faculty of Regional Sciences, Tottori University, Tottori 680-8551, Japan; ntakeda@rs.tottori-u.ac.jp (N.T.-O.); jtamura@rs.tottori-u.ac.jp (J.T.)

**Keywords:** cell fusion, chondroitin sulfate E, chondroitinase ABC, fusion index, myogenesis

## Abstract

Chondroitin sulfate (CS), a type of glycosaminoglycan (GAG), is a factor involved in the suppression of myogenic differentiation. CS comprises two repeating sugars and has different subtypes depending on the position and number of bonded sulfate groups. However, the effect of each subtype on myogenic differentiation remains unclear. In this study, we spiked cultures of C_2_C_12_ myoblasts, cells which are capable of undergoing skeletal muscle differentiation, with one of five types of CS (CS-A, -B, -C, -D, or -E) and induced differentiation over a fixed time. After immunostaining of the formed myotubes with an anti-MHC antibody, we counted the number of nuclei in the myotubes and then calculated the fusion index (FI) as a measure of myotube differentiation. The FI values of all the CS-treated groups were lower than the FI value of the control group, especially the group treated with CS-E, which displayed notable suppression of myotube formation. To confirm that the sugar chain in CS-E is important in the suppression of differentiation, chondroitinase ABC (ChABC), which catabolizes CS, was added to the media. The addition of ChABC led to the degradation of CS-E, and neutralized the suppression of myotube formation by CS-E. Collectively, it can be concluded that the degree of suppression of differentiation depends on the subtype of CS and that CS-E strongly suppresses myogenic differentiation. We conclude that the CS sugar chain has inhibitory action against myoblast cell fusion.

## 1. Introduction

Skeletal muscle is a syncytial tissue that is formed through the fusion of multiple myocytes leading to multi-nucleated myofiber bundles [1,2]. Skeletal muscle differentiation begins with activation of satellite cells located directly under the myofiber basal lamina. The satellite cells are normally in a quiescent or dormant state, but they are activated upon injury or excessive muscular burden. Once activated, the satellite cells re-enter the cell cycle, rapidly proliferate, and form numerous myoblasts. After being exposed to various stimuli, the myoblasts proceed to the differentiation stage [3,4]. During differentiation, proteins such as myosin are synthesized in order to construct myofibrils. Myoblasts that are ready for cell fusion move to fuse with other myoblasts or recently formed myotubes to become mature myotubes [5]. Owing to this cell fusion, multiple nuclei reside within a single myotube.

Chondroitin sulfate (CS), a type of glycosaminoglycan, is widely found in proteoglycans on the cell surface and throughout the extracellular matrix [6,7]. CSs are composed of two repeating sugar residues, glucuronic acid and *N*-acetylgalactosamine, and they are classified as CS-A, -B, -C, -D, -E, -K, or -H, based on the position and number of the sulfate groups [8]. CS is not only responsible for maintaining tissue structure but also plays crucial roles in developmental processes such as embryonic division and in the onset of a variety of diseases [9,10,11]. The variety of CS functions arises from the structural diversity of the repeating oligosaccharide region, including modifications by sulfate groups [11]. In recent years, studies on the process of myogenic differentiation have revealed that a decrease in CS in the culture environment acts as a trigger for myoblasts to undergo fusion to form multi-nucleated cells [12]. Therefore, it is thought that CS is a suppressor of myotube formation. Nevertheless, the effect of CS subtypes on the suppressive effects on differentiation is still unclear. In this study, we attempted to determine the type of CS that most strongly affects myotube formation.

## 2. Results and Discussion

### Suppression of Myotube Formation by CS

**Figure 1:**
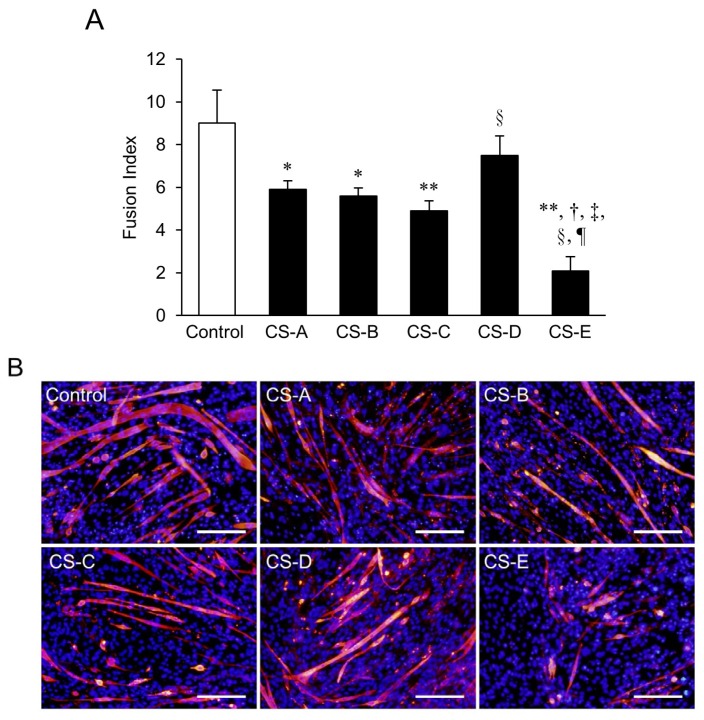
Differences in suppression of myotube formation by chondroitin sulfate (CS) subtypes. (**A**) fusion index (FI) value of C_2_C_12_ cells that were induced to differentiate for 9 days in differentiation medium supplemented with 0.2 mg/mL of each type of CS. FI values of each group were compared using the Bonferroni/Dunn *post-hoc* tests. Mean ± SE; *n* = 5. * *p* < 0.05, ** *p* < 0.01 vs. control, respectively, ^†^ *p* < 0.01 vs. CS-A, ^‡^ *p* < 0.01 vs. CS-B, ^§^ *p* < 0.05 vs. CS-C, and ^¶^ *p* < 0.01 vs. CS-D; (**B**) Fluorescent immunostaining images of CS-treated groups on Day 9 of differentiation. MHC-positive myotubes are stained red and nuclei are stained blue (Hoechst stain). The control C_2_C_12_ cells were cultured in differentiation medium without CS. A decrease in myotube length and width was observed in each CS-treated group as compared to the control. Of the five CS subtypes, the CS-E-treated cells showed the greatest decreases. Bar = 200 μm.

A decrease in the fusion index (FI) value was seen in all of the CS treated groups; in particular, groups treated with CS-A, -B, -C, and -E subtypes had significantly (*p* < 0.05) lower FI values than those in the control group (Figure 1A). Myosin heavy chain (MHC)-positive myotubes were observed in all of the groups, but the CS-E-treated group showed the most notable decrease in the length and width of MHC-positive myotubes (Figure 1B). The FI value of the CS-E-treated group was also the lowest among the groups (*p* < 0.01), and this correlated with the immunostaining results.

**Figure 2:**
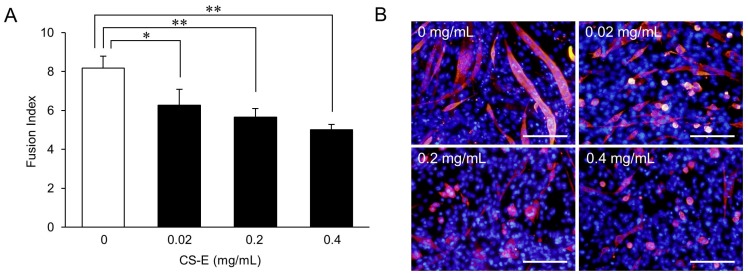
Dose-response of chondroitin sulfate E sodium (CS-E) effect on myotube formation. (**A**) FI value of C_2_C_12_ cells that were induced to differentiate for 9 days in differentiation medium supplemented with 0.02, 0.2, or 0.4 mg/mL of CS-E. FI values of each group were compared using the Bonferroni/Dunn *post-hoc* tests. Mean ± SE; *n* = 5; * *p* < 0.05, ** *p* < 0.01; (**B**) Fluorescent immunostaining images of C_2_C_12_ cells treated with 0.02, 0.2, or 0.4 mg/mL of CS-E on Day 9 of differentiation. MHC-positive myotubes are stained red, and nuclei are stained blue (Hoechst stain). Control C_2_C_12_ cells were cultured in differentiation medium with vehicle lacking CS-E (0 mg/mL). Bar = 200 μm.

A dose-response curve for the effect of CS-E on myotube formation (Figure 2A) showed that the FI value of the 0.02 mg/mL CS-E-treated group was significantly (*p* < 0.05) lower than that of the control group (0 mg/mL) and that the FI values of the 0.2 mg/mL and 0.4 mg/mL CS-E-treated groups showed further concentration-dependent decreases (*p* < 0.01) compared with the FI value of the control group. A decrease in myotube length and width was observed in the 0.02 mg/mL CS-E-treated group as compared to the control (Figure 2B). Furthermore, non-elongated myotubes were increased in the 0.2 mg/mL and 0.4 mg/mL CS-E-treated groups. To form a mature myotube, myoblast fusion starts with cell elongation, followed by migration, cell-to-cell recognition and adhesion, and finally ends with membrane fusion [5,13,14]. Despite being MHC-positive by immunostaining, the thin, non-elongated myotubes led us to infer that CS, which is a known suppressor of myotube formation, suppressed myotube formation at the initial step of cell fusion, and that CS-E is the strongest suppressor among CSs.

**Figure 3:**
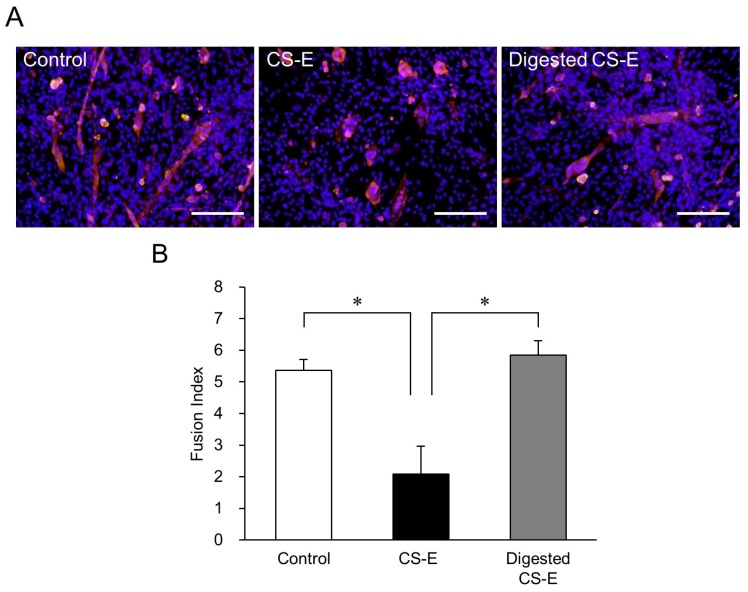
Effects of CS-E digestion by chondroitinase ABC (ChABC) on myotube formation. (**A**) Fluorescent immunostaining image of C_2_C_12_ cells after inducing differentiation for 9 days with differentiation medium without CS-E (control group), differentiation medium containing 0.2 mg/mL CS-E, and differentiation medium containing 0.2 mg/mL ChABC-digested CS-E. MHC-positive myotubes are stained red and nuclei stained blue (Hoechst stain). The undigested CS-E-treated cells had fewer elongated myotubes, whereas long myotubes were observed in the cells treated with ChABC-digested CS-E. Bar = 200 μm; (**B**) FI values of C_2_C_12_ cells that were induced to differentiate for 9 days in differentiation medium containing either CS-E or ChABC-digested CS-E. The control group was cultured in differentiation medium without CS-E. FI values of each group were compared using the Bonferroni/Dunn *post-hoc* tests. Mean ± SE; *n* = 5; * *p* < 0.05.

Compared with the control group, the CS-E-treated group had very few long MHC-positive myotubes, whereas, in contrast, the group treated with CS-E digested by chondroitinase ABC (ChABC) resembled the control group (Figure 3A). In parallel, the FI value of the undigested CS-E-treated group was significantly lower than that of the control group (*p* < 0.05), but the FI value of the digested CS-E-treated group was as high as that of the control group (Figure 3B). Since the CS-E mediated suppression of myotube formation was abolished by ChABC digestion, this led us to conclude that the long chain structure of CS-E is important for suppression.

The basic backbone of CS possesses two alternating sugar residues: glucuronic acid and *N*-acetylgalactosamine. Over-sulfated types of CS, such as CS-D and -E, which have bi-sulfated sugar units, have been identified in various tissues and cells of invertebrates, marine vertebrates, and higher land vertebrates, and possess a unique functional domain [15]. Numerous studies have suggested that CS-E acts on growth factors, cytokines, extracellular matrix proteins, and various other bioactive proteins [8,16,17,18]. In this study, although suppression of myotube formation was seen for each CS subtype, the degree of suppression varied. The suppression effects of CS-E were especially prominent. These results indicate that CS-E, an oversulfated CS subtype, strongly interacts with certain types of bioactive proteins during myotube formation. It has been reported that when myoblasts fuse, a muscle-specific cell membrane protein, TMEM8C (known as myomaker), is expressed on the cell surface [19,20]. In this experiment, MHC-positive thin, non-elongated, myotubes were observed in the CS-E-treated group. This led us to believe that one possible mechanism for suppression of myotube formation involves CS-E acting on myomaker, thereby inhibiting cell membrane fusion between neighboring myoblasts.

Thus, our results show that in C_2_C_12_ cells, the degree of suppression of differentiation depends on the CS subtype, and of the subtypes tested, CS-E suppressed myogenesis the greatest. In addition, our data suggest that sugar chain structure plays a key role in the inhibition of myogenesis. We believe that the present results provide additional novel insights into understanding the relationship between muscle histogenesis and proteoglycans and that removal of CS from damaged muscular tissue may be an effective therapeutic strategy to achieve muscle regeneration.

## 3. Materials and Methods

### 3.1. Cell Culture and Induction of Myogenic Differentiation

C_2_C_12_ mouse myoblast cells, which are capable of undergoing skeletal muscle differentiation, were obtained from the RIKEN BioResource Center (Tsukuba, Japan) and cultured in Dulbecco’s modified Eagle’s medium (DMEM; Invitrogen-Gibco, Palo Alto, CA, USA) with 10% fetal bovine serum (FBS; Atlas Biologicals, Fort Collins, CO, USA). C_2_C_12_ cells (2 × 10^5^ cells/mL) were seeded in each well of a 12-well plate, and then incubated at 37 °C and 5% CO_2_ in a CO_2_ incubator until 80%–90% confluent.

DMEM with 2% horse serum (Sigma-Aldrich, St. Louis, MO, USA) was used as the differentiation medium. The day on which the medium was changed from DMEM containing 10% FBS to the differentiation medium was designated as the start of differentiation, Day 0.

### 3.2. Immunocytochemistry

C_2_C_12_ cells were fixed in 10% formalin neutral buffer solution (Wako, Osaka, Japan) for 10 min at room temperature. Cells were washed with 10 mM phosphate buffered saline (PBS), and the reaction with PBS and 0.5% Triton X-100 (Wako) was allowed to proceed for 20 min for permeabilization. After washing again with PBS, blocking solution containing donkey serum (Gene Tex, Irvine, CA, USA) was added for 20 min. Cells were then incubated at room temperature for 90 min with an anti-mouse MHC antibody (R&D, Minneapolis, MN, USA) that had been diluted to 1:50 with PBS (final concentration 10 μg/mL) at room temperature. After washing, cells were incubated for 20 min with a 1:200 dilution of NL-557-labeled anti-mouse IgG donkey antibody (R&D), and then, the nuclei were stained with 5 μg/mL Hoechst 33342 stain (Molecular Probes, Eugene, OR, USA). The stained cells were observed under an OLYMPUS U-TBI90 fluorescence microscope (Tokyo, Japan). Negative controls, in which the primary antibodies were replaced with non-immunized serum, did not show any staining.

### 3.3. Assessment of Muscle Differentiation

A myotube was defined as having three or more nuclei from fused cells and being MHC-positive. Five arbitrary visual fields were selected per replicate sample (*n* = 5 replicates) giving a total of twenty-five visual fields per group. The number of nuclei inside of myotubes was manually counted. The FI, which was used as the myogenic differentiation index, was found by averaging the number of nuclei inside the myotubes.

### 3.4. Identification of CS Subtypes Affecting Differentiation

From Day 0 to Day 9, differentiation was induced with the differentiation medium containing 0.2 mg/mL CS. Five types of CS preparations dissolved in PBS were used in this study—chondroitin sulfate A sodium salt (CS-A; derived from whale cartilage), chondroitin sulfate B sodium salt (CS-B; derived from pig skin), chondroitin sulfate C sodium salt (CS-C; derived from shark cartilage), chondroitin sulfate D sodium salt (CS-D; derived from shark fin), and chondroitin sulfate E sodium salt (CS-E; derived from Japanese common squid (*Todarodes pacificus*)) (all from PG Research, Tokyo, Japan). The CS-E preparation used in this study contained CS-A, -C, -D, -E and chondroitin (non-sulfated) at 18.0%, 5.7%, 0.1%, 67.3%, and 5.1%, respectively (CS-E data sheet from PG Research). The differentiation medium containing each CS was replaced with fresh CS-containing medium every three days to maintain optimal culture conditions. After 9 days of inducing differentiation, the FI value was calculated by immunofluorescence staining with anti-MHC antibodies and Hoechst 33342. The vehicle control group was cultured for 9 days in differentiation medium without any CS.

### 3.5. Dose-Response Effects of CS-E

As CS-E, out of the five subtypes, demonstrated the strongest suppression on myotube differentiation, we spiked the differentiation medium with 0.02, 0.2, or 0.4 mg/mL of CS-E, and then calculated the FI value for each group on Day 9.

### 3.6. Analysis of the Effects of the CS-E Glycan Region on Myotube Formation

ChABC catabolizes CS and degrades CS-E to its smallest unit, the disaccharide, which lacks almost any physiological activity [21]. We investigated whether or not catabolizing CS-E would neutralize the suppression of myotube formation that was observed upon addition of CS-E. To degrade CS-E, equal volumes of 10 mg/mL CS-E and 1 U/mL ChABC (Sigma-Aldrich) were mixed and then allowed to react overnight at 37 °C. Cells were cultured for 9 days in the differentiation medium containing 0.2 mg/mL digested CS-E, and the FI value was determined by immunostaining using anti-MHC antibody. For comparison, a control group was cultured in differentiation medium without CS-E, and a positive control group was cultured in differentiation medium with 0.2 mg/mL undigested CS-E.

### 3.7. Statistical Analysis

Statistical significance of the values obtained from each experiment was evaluated by one-way analysis of variance (ANOVA) and Bonferroni/Dunn *post-hoc* tests. *p* values of less than 0.05 were considered statistically significant.

## 4. Conclusions

We have found that CS inhibited cell fusion during the course of myogenic differentiation, and of the tested subtypes, CS-E was the strongest suppressor. Furthermore, the structure of CS-E’s sugar chain is thought to be deeply involved in the suppression of myotube formation.
